# A quantitative and precision‑oriented neuronal reconstruction approach based on data grading

**DOI:** 10.1186/s40708-026-00314-0

**Published:** 2026-06-23

**Authors:** Mingwei Liao, Chi Xiao, Xiaojun Wang, Qingming Luo, Hui Gong, Anan Li

**Affiliations:** 1 MOE Key Laboratory for Biomedical Photonics, Wuhan National Laboratory for Optoelectronics, Huazhong University of Science and Technology, Wuhan, 430074 China; 2 HUST-Suzhou Institute for Brainsmatics, JITRI, Suzhou, 215123 China; 3 Key Laboratory of Biomedical Engineering of Hainan Province, School of Biomedical Engineering, Hainan University, Sanya, 572025 China

**Keywords:** Neuron reconstruction, Match reconstruction, Allocate reconstruction, The model of neuron reconstruct, Precision reconstruction

## Abstract

Accurate and efficient neuronal reconstruction is essential for large-scale neuronal projection analysis and neural circuit mapping. However, conventional reconstruction approaches are often constrained by the structural complexity of neurons, the diversity of imaging signals, and variations in annotator expertise, making it difficult to simultaneously achieve high reconstruction quality and efficiency. To address these challenges, this study proposes a quantitative and precision-oriented neuronal reconstruction framework that systematically integrates reconstruction efficiency and accuracy modeling, data–algorithm matching, and refined task allocation strategies. First, mathematical models were established to quantitatively characterize reconstruction efficiency and accuracy, providing a theoretical foundation for precision reconstruction. Based on quantitative indicators of neuronal reconstruction difficulty, a data–algorithm precise matching strategy was developed to adaptively select the most suitable reconstruction method for different types of neuronal data while leveraging the complementary strengths of multiple reconstruction algorithms. Experimental results demonstrated significant improvements in reconstruction accuracy across multiple data categories, with the best-performing image category achieving an accuracy improvement of up to 18.8%. Furthermore, a data–annotator precise allocation strategy was proposed to match data difficulty with annotator capability, enabling efficient human–machine collaborative reconstruction and transforming conventional experience-based reconstruction into a precision-driven quantitative reconstruction paradigm. Compared with traditional reconstruction strategies, the proposed allocation strategy improved reconstruction accuracy by 44.3% and increased overall reconstruction efficiency by 34.6%. In summary, the proposed framework enables quantitative evaluation and controllable assurance of neuronal reconstruction quality. By transforming neuronal reconstruction from a conventional single-method paradigm into a data-driven precision decision-making paradigm, the proposed approach substantially improves reconstruction efficiency while maintaining high reconstruction quality. This work provides reliable methodological support and a solid data foundation for large-scale neuronal morphology analysis and neural circuit research.

## Introduction

Neurons are the fundamental structural and functional units of the brain. Their morphological characteristics not only encode the structural information of individual cells but also largely determine the connectivity patterns and functional properties of neural circuits. The spatial distribution, branching patterns, trajectories of dendrites and axons, as well as their connection sites with other cells, collectively constitute the structural basis for neural information processing [1]. Therefore, achieving complete and high-precision neuronal morphology reconstruction is of irreplaceable importance for understanding the mechanisms of brain information encoding, the implementation of cognitive functions, and the generation of complex behaviors [2, 3].

The development of high-resolution imaging technologies has provided critical support for this goal. Next-generation optical imaging platforms such as fMOST enable continuous imaging of mouse brains and even larger-scale brain tissues at submicron resolution, thereby capturing fine structural details of complete neurons [4]. However, these imaging technologies generate extremely large datasets. The three-dimensional whole-brain imaging data of an adult mouse can reach tens to hundreds of terabytes [5, 6]. Under such circumstances, how to efficiently and accurately accomplish three-dimensional neuronal reconstruction from massive datasets has become one of the central challenges in neuroscience.

Large-scale neuronal reconstruction is not merely a technical challenge but a highly complex systems engineering problem [7, 8]. First, neuronal imaging data exhibit significant complexity and heterogeneity. Fiber structures, signal intensity, and fiber density vary substantially across different brain regions [9–12]. Second, neuronal morphology spans extensive spatial scales. Axons and dendrites may extend from hundreds of micrometers to several millimeters or even longer, often crossing multiple brain regions and hierarchical structures, further increasing reconstruction difficulty [13, 14]. Although manual tracing methods offer high accuracy, their efficiency is extremely limited. Fully automated reconstruction methods significantly improve efficiency but struggle to maintain stable reconstruction quality under complex data conditions [15, 16]. These factors restrict the balance between scalability and fidelity in neuronal reconstruction.

To alleviate these challenges, various reconstruction strategies have been proposed. From early manual annotation tools [17] to recent advances in automated reconstruction algorithms [18, 19], progress has been made in accelerating neuronal reconstruction; however, achieving both high quality and high efficiency remains difficult. Consequently, human–machine collaborative reconstruction has attracted increasing attention. In this paradigm, automated algorithms generate initial reconstructions that are subsequently refined through manual correction. Tools such as Vaa3D [20] and GTree [21] partially balance efficiency and accuracy. Nevertheless, these approaches remain highly dependent on data complexity and operator expertise, limiting their scalability for truly large-scale, high-quality reconstruction. Moreover, in conventional workflows, large datasets are often simply partitioned and evenly assigned to human annotators or algorithms. This uniform processing strategy neglects intrinsic data heterogeneity, potentially increasing error rates and degrading overall reconstruction quality. In other words, without explicit modeling and grading of reconstruction difficulty, both efficiency and quality are fundamentally constrained [22, 23].

To address these limitations, this study proposes a quantitative precision-driven neuronal reconstruction framework based on data grading. First, focusing on the core issues in the reconstruction process, we establish accuracy and efficiency models to quantitatively describe and analyze reconstruction performance, providing a theoretical foundation for the design of precision reconstruction strategies. Based on these models, we systematically evaluate the influence of multiple factors on reconstruction quality and efficiency, identify dominant factors, and optimize the reconstruction workflow accordingly. Building upon the image difficulty grading system proposed by Liao et al. [23], we develop a difficulty-aware data classification method and precisely match each data category with its optimal automated reconstruction algorithm. This strategy significantly improves both overall and category-specific reconstruction accuracy, with performance gains of up to 18.8% for certain data types. Furthermore, for graded neuronal datasets, we design a precision task allocation model that assigns data of varying difficulty to reconstruction personnel with corresponding expertise, maximizing the utilization efficiency of human and computational resources while ensuring reconstruction accuracy. Experimental results demonstrate that, compared with conventional reconstruction strategies, the proposed precision allocation framework improves reconstruction accuracy and overall efficiency by 44.3% and 34.6%, respectively. In summary, the proposed quantitative precision-driven neuronal reconstruction framework substantially enhances both accuracy and efficiency in large-scale neuronal reconstruction, providing a reliable theoretical foundation and practical pathway toward high-quality whole-brain neuronal morphology reconstruction.

**Fig. 1:**
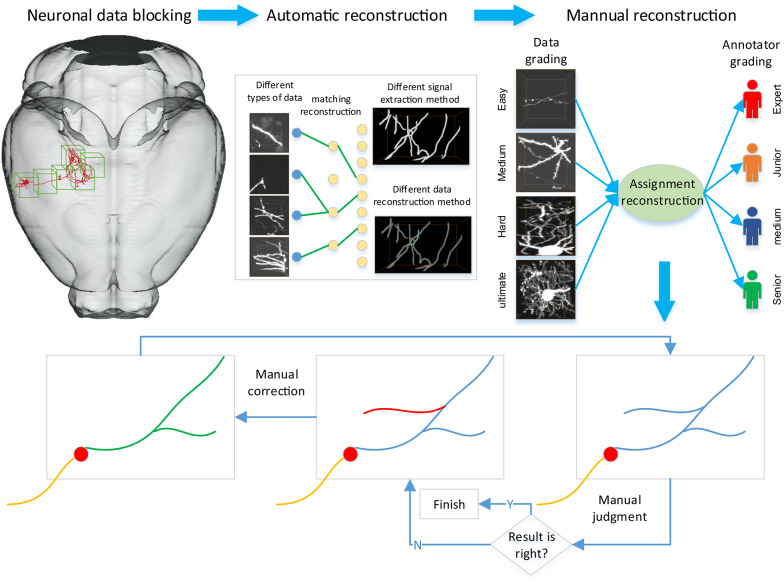
Precision neuronal reconstruction framework

## Methods

### Neuron reconstruction main framework

To achieve precise neuronal reconstruction, the overall workflow consists of three key stages, as illustrated in Fig. 1.

First, a neuron block segmentation strategy is employed to divide the original imaging data containing multiple neurons into independent image blocks. This enables parallel processing of individual data blocks and significantly improves the overall reconstruction efficiency. After segmentation, each image block is quantitatively evaluated and graded according to the image difficulty assessment method proposed by Liao et al. [23], forming datasets with different difficulty levels that provide a foundation for subsequent reconstruction strategies.

Subsequently, image blocks are further categorized based on the grading indicators. Considering the performance variations of different automatic reconstruction algorithms across diverse data types, a precise matching strategy for automated reconstruction is constructed. Specifically, for each category of image blocks, the optimal combination of signal extraction methods and neuronal reconstruction algorithms is selected to enhance the accuracy and robustness of automatic reconstruction under complex data conditions.

After obtaining the automatic reconstruction results, differences in structural complexity and reconstruction difficulty among neuronal data blocks, as well as variations in the ability and experience of participating annotators, are taken into account. A precision task allocation mechanism based on data difficulty and personnel capability is therefore designed. Data of different difficulty levels are assigned to annotators with corresponding competency levels. The standard manual reconstruction procedure includes two steps–manual evaluation and manual correction–to complete the final neuronal reconstruction.

Through this precision-oriented reconstruction framework, the overall reconstruction efficiency is significantly improved while maintaining high reconstruction quality, thereby achieving a coordinated optimization of accuracy and efficiency.

### Establishment of neuronal reconstruction efficiency model

To effectively improve the quality and efficiency of neuronal reconstruction, establishing quantitative models for reconstruction quality and reconstruction efficiency is fundamental for accurately analyzing the neuronal reconstruction process and enabling quantitative optimization.

Based on an analysis of the neuronal reconstruction workflow, the factors influencing reconstruction evaluation metrics mainly consist of two core components. The first component is automatic neuronal reconstruction, and the second component is manual reconstruction. Manual reconstruction includes two primary steps: manual evaluation and manual correction. Manual evaluation refers to the process in which one or multiple annotators determine whether the reconstruction result of a data block is correct or incorrect, and this process continues until the reconstruction result of the image block satisfies the required accuracy criteria. When the reconstruction result of an image block is incorrect, manual correction is required; when the reconstruction result is correct, the reconstruction process for that image block is completed. Manual correction mainly involves a single annotator modifying incorrectly reconstructed image blocks until the reconstruction result is corrected. After the correction is completed, further manual evaluation is required, and this iterative process continues until the reconstruction result is confirmed to be correct.

Therefore, the time consumption of reconstruction can be divided into computational time and manual time. Throughout the entire reconstruction process, since the reconstruction workflow operates in a pipeline manner, automatic computation and manual operations are conducted simultaneously. Moreover, computational efficiency can be improved by increasing the number of distributed computing nodes and the volume of reconstruction data, thereby enhancing computational throughput. Consequently, reconstruction efficiency is primarily determined by manual time consumption. During the reconstruction process, manual time consumption mainly arises from manual evaluation of reconstructed data blocks and manual correction of incorrectly reconstructed data. Based on this analysis, the following neuronal reconstruction efficiency model is established:

1 $$\begin{aligned} V = \frac{L}{{\sum \limits _{i = 1}^n {\sum \limits _{j = 1}^m {{x_{ij}} \cdot {t_{ij}}} } + \sum \limits _{l = 1}^{\bar{n}} {\sum \limits _{v = 1}^m {{y_{lv}} \cdot {t_{lv}}} } }} \end{aligned}$$

Here, *L* denotes the total length of the *n* image blocks. $x_{ij}$ indicates whether annotator *j* processes image block *i*, and $t_{ij}$ represents the time taken by annotator *j* to judge image block *i*. *m* denotes the total number of annotators. $\bar{n}$ represents the number of image blocks among the *n* blocks that require correction tasks. $y_{lv}$ indicates whether user *v* processes image block *l*, and $t_{lv}$ denotes the time taken by user *v* to correct image block *l* we generally avoid assigning the same image block to the same person more than once, therefore, $x_{ij}$ and $y_{lv}$ take binary values of 0 or 1.

### Establishment of neuronal reconstruction accuracy model

During the reconstruction process, our analysis shows that each image block first undergoes automatic reconstruction, where the accuracy of the automatic reconstruction primarily determines the initial probability that the image block is correctly reconstructed. The reconstruction difficulty of an image block affects its overall reconstruction accuracy by influencing both the automatic reconstruction accuracy and the manual reconstruction accuracy. Therefore, the reconstruction accuracy of image blocks with different difficulty levels can be directly analyzed in terms of automatic reconstruction accuracy and personnel reconstruction accuracy. In the manual evaluation stage, the number of annotators involved and their individual accuracy play a decisive role in determining the final reconstruction accuracy of an image block. This is because manual evaluation serves as the entry point for submitting the final reconstruction result in the entire workflow. Only when a consensus is reached in this stage will the reconstruction result be accepted and further extended.

In the manual correction stage, since only one annotator is assigned to correct each image block, the reconstruction accuracy at this stage depends solely on the correction accuracy of that annotator. After correction is completed, the image block must re-enter the manual evaluation stage to determine whether the reconstruction is correct. This process iterates until the reconstruction result is confirmed to be correct.

Based on the above analysis, we summarize the core issues of the model as follows. First, the initial probability that an image block is correctly reconstructed is denoted as ${P_0}$ which corresponds to the accuracy of automatic reconstruction. Then, during the manual evaluation process, the number of annotators is gradually increased to ${n^k}$ until the posterior confidence (accuracy) of the group decision exceeds a predefined threshold ${A_t}$ If the evaluation result is judged to be correct, the process stops, and the posterior probability at that round is taken as the final estimate of correctness. Otherwise, if the evaluation result is judged to be incorrect, the process enters the correction stage, where the probability that the reconstruction becomes correct after correction is denoted as ${P_{k + 1}} = {P_k} + (1 - {P_k}) \cdot c$. Finally, the manual evaluation and manual correction procedures are repeated iteratively until the reconstruction result is confirmed to be correct.

According to the above analysis, we establish a set of iterative model equations. Let ${P_k}$ denote the probability that the reconstruction result is correct at the *k* round of the task. After evaluation by multiple annotators, the probability that the group decision result is correct can be expressed as:

2 $$\begin{aligned} {J_{true}}({n^k}) = \prod \limits _{v \in {\phi _k}} {{p_v}\prod \limits _{r \in {\varphi _k}} {(1 - {p_r})} } \end{aligned}$$

The probability that the result is incorrect after multi-person judgment is calculated as follows:

3 $$\begin{aligned} {J_{false}}({n^k}) = \prod \limits _{v \in {\phi _k}} {(1 - {p_v})\prod \limits _{r \in {\varphi _k}} {({p_r})} } \end{aligned}$$

Here, $\left| {{\phi _k}} \right| + \left| {{\varphi _k}} \right| = {n^k}$ denotes the total number of participants involved in the judgment at round *k*. $\left| {{\phi _k}} \right|$ denotes the number of participants who judge the result as correct, and $\left| {{\varphi _k}} \right|$ denotes the number of participants who judge the result as incorrect. ${p_v}$ and ${p_r}$ represent the judgment accuracy of different individuals.

During the correction process, the modification is applied directly to the image block itself. Therefore, in the next iteration, the accuracy of the image block depends only on the probability of successful manual correction and is independent of the accuracy in the previous round. The update formula for the data accuracy in the correction stage is given as follows:

4 $$\begin{aligned} {P_k} = {c_k} \end{aligned}$$

That is, during neuron image block correction, each image block can only be treated as an integral unit, and it is not possible to modify erroneous parts independently. Here, ${c_k}$ represents the correction accuracy of the operator, the probability that the reconstructed result of the image block is correct after manual correction.

Finally, according to Bayes’ theorem, the posterior probability of the image block after the judgment result in the *k* round is given by:

5 $$\begin{aligned} {P_{k + 1}} = \frac{{{P_k} \cdot {J_{true}}({n^k})}}{{{P_k} \cdot {J_{true}}({n^k}) + (1 - {P_k}) \cdot {J_{false}}({n^k})}} \end{aligned}$$

Its computational form remains unchanged. Through this model, both stages are fully incorporated into a unified probabilistic accuracy framework, which provides a quantitative foundation for subsequent quantitative analysis and image block allocation.

### Establishment of a precise matching strategy for automatic neuronal data reconstruction methods

Given the significant differences in reconstruction accuracy across various types of image blocks and different automatic reconstruction algorithms, this study aims to construct an optimal matching relationship among image block type–signal extraction method–reconstruction algorithm, in order to maximize reconstruction accuracy under given conditions.

To achieve precise matching between image blocks and reconstruction methods, we first quantify the reconstruction difficulty of neuronal image blocks based on the grading method proposed by Liao et al. [23]. The grading framework consists of the following steps. First, a set of difficulty-related quantitative indicators is selected, including 11 features: Fiber density, Target fiber density, Key point density, Target key point density, Fiber length, Target fiber length, Number of key points, Number of target key points, Signal density, Projection signal density, and Weak signal ratio. These indicators are categorized into three groups: fiber-related, signal-related, and key-point-related features.

Next, we performed quantitative feature extraction on the images to evaluate the reconstruction difficulty of neuronal data blocks. For fiber-related metrics, we first applied the principal curve method to extract the fiber skeleton, and the fiber length was computed based on the distances between skeleton points. The corresponding fiber density was quantified by dividing the total skeleton length by the maximum area among the three orthogonal views of the data.

For key-point-related metrics, the feature sphere method was used to extract key-point coordinates. The number of key points was directly obtained by counting detected points, and the key-point density was measured as the number of key points divided by the maximum area across the three views.

For signal-related metrics, an adaptive thresholding method was first applied to extract fiber signals. Signal density was calculated as the number of three-dimensional signal points divided by the maximum area of the three orthogonal views. The projected signal density was obtained by projecting the three-dimensional signals onto the three orthogonal planes, and taking the minimum signal density across the three views as the final metric.

After obtaining the quantitative values of the above 11 indicators, principal component analysis (PCA) was applied to reduce dimensionality and eliminate redundant information. Based on a cumulative explained variance threshold of 90%, four principal components were retained. These components were then used to compute a difficulty coefficient for each data block. Subsequently, hierarchical clustering was employed to construct a dendrogram, and the data were finally divided into four difficulty levels according to the resulting clustering tree.

Through this procedure, both the quantitative difficulty indicators and the corresponding difficulty levels of the image blocks are obtained. After obtaining the difficulty metrics for each data sample, it is necessary to further analyze the quantitative relationship between the difficulty indicators and automatic reconstruction accuracy.

For neuronal data, multiple signal extraction methods are first employed for preprocessing, including Otsu thresholding [24], VoxResNet [25], and nnU-Net [26]. Among them, Otsu thresholding effectively preserves structural features of the original image. VoxResNet performs well in weak-signal image processing but exhibits relatively lower stability in high-density fiber regions. nnU-Net demonstrates strong generalization capability in overall signal extraction. To fully exploit the complementary advantages of these methods, all three signal extraction approaches are adopted in this study. Together with the original image data, four types of preprocessing results are generated for subsequent reconstruction experiments.

After obtaining different preprocessing results, multiple automatic reconstruction algorithms are applied to reconstruct neuronal images, including NeuroGPS-Tree [27], APP2 [18], neuTube [28], PHD [29], SmartTracing [19], CAAT [30], PointTree [31], SparseTracer [32], and ST-LFV [33]. Since this study focuses on single-neuron reconstruction, whereas some algorithms are primarily designed for multi-neuron image blocks, post-processing is required after reconstruction. Specifically, based on a predefined starting point, the nearest reconstructed node in the result is identified and used as the truncation starting point. Then, according to the initial growth direction, only the reconstruction branches consistent with the target neuron are retained to obtain the single-neuron reconstruction result. Finally, reconstruction results from each algorithm are compared with the gold standard, and the corresponding F1 scores are calculated. Results with F1 $\ge$ 0.99 are regarded as correct reconstructions. The filtered data are subsequently used for analyzing and constructing the matching relationships.

During the indicator selection process, we conducted a correlation analysis on the initially selected 11 image feature indicators. It was observed that some indicators are highly correlated in terms of physical meaning and representational capability; for example, fiber density and key point density both reflect the spatial concentration of signals to a certain extent. When the correlation coefficient between two indicators exceeds 0.8, they are considered to belong to the same category. Therefore, during feature selection, only the indicator with the most significant impact on reconstruction accuracy within each category is retained, while those with weaker influence are removed.

Subsequently, a traversal-based strategy is used to set threshold values for each retained indicator in ascending order, and image blocks are classified into different categories according to these thresholds. For each category, the optimal “signal extraction method–reconstruction algorithm” combination is selected. The improvement in reconstruction accuracy achieved by the proposed classification-and-matching strategy, compared with the single best reconstruction method, is then calculated. Finally, the two indicators with the largest improvement are retained for final image block classification. For these two indicators, the optimal thresholds are again determined using the traversal method, thereby enabling precise matching between different types of image blocks and reconstruction methods.

In summary, by constructing a precise matching framework among image block types, signal extraction methods, and reconstruction algorithms, this study effectively improves the overall accuracy of automatic neuronal reconstruction and provides a solid methodological foundation for achieving efficient, stable, and scalable large-scale neuronal reconstruction.

### Data-driven precise task allocation-based neuronal reconstruction method

To quantitatively guarantee neuronal reconstruction accuracy and efficiency, reconstruction tasks must be reasonably allocated among participating personnel. Owing to the variability in reconstruction difficulty across image blocks and the heterogeneity in individual reconstruction abilities, achieving precise matching between image blocks and personnel is a critical prerequisite for ensuring reconstruction accuracy. Reconstruction quality and efficiency constitute the two most fundamental objectives of the neuronal reconstruction process. In traditional manual reconstruction approaches, a single operator typically completes the reconstruction of an entire neuron independently. However, such a strategy struggles to balance quality and efficiency: it is prone to errors when handling complex data and simultaneously limits overall throughput. To address these limitations, this study adopts a “divide-and-conquer” strategy, in which whole neurons are decomposed into smaller data blocks. The reconstruction difficulty of each block and the reconstruction capability of each participant are then evaluated separately, followed by optimized task allocation to achieve rational matching between data and personnel.

During image block allocation, several core issues must be addressed. First, the reconstruction accuracy of each image block must be ensured to guarantee the correctness of the overall neuronal reconstruction, as validated in the preceding quantitative analysis. Second, the reconstruction process should be completed as rapidly as possible to maximize overall throughput. Meanwhile, idle waiting time for reconstruction personnel should be avoided to maintain sustained engagement and motivation. Furthermore, from a system operation perspective, it is desirable to minimize both the total number of participating personnel and the overall working time in order to reduce system operating costs. Based on these considerations, an optimization model is established to address the task allocation problem. In the model, the known information includes the difficulty level of each image block, the ability level and operational accuracy of reconstruction personnel, the number of online personnel at each level, and the number of image blocks at each difficulty level. Based on the analysis, the model must satisfy the following constraints.

First, the reconstruction accuracy of each image block must meet the predefined minimum requirement. Second, the accuracy of personnel participating in the reconstruction of a given image block must not be lower than the minimum accuracy threshold required for that block. Finally, the personnel level assigned to an image block must not be lower than the difficulty level of the image block itself. The corresponding mathematical constraints can be formulated as the following three inequalities:

6 $$\begin{aligned} \begin{array}{l} s.t.\mathrm{ }{A_j} \ge {A_t}\\ \mathrm{ }{r_i} > {r_t}\\ \mathrm{ }{l_i} \ge {l_j} \end{array} \end{aligned}$$

Here, ${A_j}$ represents the accuracy of an image block. According to the accuracy model, its calculation formula is as follows:

7 $$\begin{aligned} & {A_j} = \frac{{\prod \limits _{v \in {\phi _j}} {{r_v}\prod \limits _{p \in {\varphi _j}} {(1 - {r_p})} } }}{{\prod \limits _{v \in {\phi _j}} {{r_v}\prod \limits _{p \in {\varphi _j}} {(1 - {r_p})} } + \prod \limits _{v \in {\phi _j}} {(1 - {r_v})\prod \limits _{p \in {\varphi _j}} {({r_p})} } }}, \nonumber \\ & ({x_{vj}} = 1,{x_{pj}} = 1) \end{aligned}$$

Here, ${A_t}$ denotes the minimum accuracy requirement for an image block, and ${r_t}$ denotes the minimum accuracy required for personnel assigned to that image block. ${r_i}$, ${r_v}$ and ${r_p}$ represent the accuracy of individual users. ${l_i} > = {l_t}$ indicates that the skill level of a user must be higher than the difficulty level of the image block. ${\phi _j}$ denotes the set of users whose work on the image block is correct, and ${\varphi _j}$ denotes the set of users whose work on the image block is incorrect.

We consider four indicators as optimization objectives: personnel waiting time, image block completion time, number of assignments per person, and the total system processing time. Regarding system processing time and personnel waiting time, if the system processing time is optimized, the personnel waiting time should also be optimized; therefore, the two can be unified. The calculation formulas for the four objective functions are as follows:

8 $$\begin{aligned} \begin{array}{l} \mathop {\min }\limits _{{x_{ij}}} {T_a} = \mathop {\max }\limits _{1 \le i \le m} \left\{ {\sum \limits _{j = 1}^n {{a_{ij}}{x_{ij}}} } \right\} \\ \mathop {\min }\limits _{{x_{ij}}} {T_u} = \sum \limits _{i = 1}^m {({T_a} - \sum \limits _{j = 1}^n {{a_{ij}}{x_{ij}}} )} \\ \mathop {\min }\limits _{{x_{ij}}} N = \sum \limits _j^n {\sum \limits _i^m {{x_{ij}}} } \\ \mathop {\min }\limits _{{x_{ij}}} {T_g} = \sum \limits _{i = 1}^m {\sum \limits _{j = 1}^n {{a_{ij}}{x_{ij}}} } \end{array} \end{aligned}$$

${T_a}$ represents the completion time for processing the data, *N* represents the total number of assignments, ${T_u}$ represents the personnel waiting time, and ${T_g}$ represents the total system processing time. ${a_{ij}}$ denotes the time required for person *i* to process image block *j*. ${x_{ij}}$ indicates whether person *i* is assigned to image block *j*, taking a value of 0 or 1.

The multi-objective task allocation problem investigated in this study involves assigning *n* tasks to *m* personnel, where each task can be collaboratively completed by multiple personnel, and each person can handle multiple tasks. Under the constraint that the total accuracy of each task meets or exceeds the threshold ${A_t}$, the objectives are to simultaneously minimize the task completion time, the total system processing time, and the total number of assignments. Since this problem is NP-hard, obtaining the exact optimal solution directly is computationally intractable. For such 0-1 multi-objective optimization problems, heuristic algorithms offer significant advantages. They can avoid exhaustive enumeration of the entire solution space and, by leveraging local information or the problem structure to guide the search, efficiently find locally optimal or near-optimal solutions. In addition, for multi-objective problems, optimal solutions are sequentially achieved by establishing target priorities.

Among all heuristic algorithms, the greedy algorithm is particularly well-suited for this type of data allocation problem. In the experimental setup, 10,000 data segments of varying difficulty levels were allocated according to their actual proportional distribution. Reconstruction times were randomly generated following a normal distribution based on empirical mean processing times and standard deviations. Worker accuracy was randomly assigned under the constraint of a minimum accuracy requirement of 0.998. The number of participating workers was sequentially set to [210, 255, 300, 360, 405] to compare the performance of different solution methods under varying personnel scales. The maximum number of workers assigned to each task was limited to five. If the task accuracy still failed to reach the predefined threshold after reaching the maximum number of workers, the task was upgraded and reassigned to a new set of workers. Under these constraints, the greedy algorithm then selected the most suitable workers based on their task completion times and accuracy, continuing this process until all data segments were fully reconstructed.

### Evaluation of the precision-assigned reconstruction method results

To analyze the influence of various factors on neuronal reconstruction accuracy and efficiency, as well as their underlying relationships, we compared precision-assigned neuronal reconstruction with conventional reconstruction using controlled variables, and simulated the neuronal reconstruction process based on graded real reconstruction data. In the precision reconstruction simulation, the process consists of five main steps. First, the image blocks to be reconstructed are arranged in an order extending from the soma. Next, each image block is traversed and classified as correct or incorrect based on the actual automated reconstruction results. Then, each image block undergoes human evaluation, where the judgment of each participant is processed through the accuracy model to obtain the final evaluation of the block.

For correctly reconstructed blocks, if the final judgment is correct, the block is marked as completed with a correct result, and reconstruction proceeds to subsequent blocks; if the judgment is incorrect, a correction operation is performed, followed by reevaluation, and this loop continues until the result is correct. For incorrectly reconstructed blocks, if the judgment is correct, a correction is applied first, followed by reevaluation in a similar loop. If the judgment is incorrect, the block is marked as completed with an incorrect result. In cases of incorrect results, a branch within the block’s reconstructed fibers is randomly selected as the error branch, and a random number is generated: if the number is greater than 0.5, the branch is designated as an over-tracing branch, and all subsequent tracing results are considered over-traced; if the number is less than or equal to 0.5, the branch is designated as an under-tracing branch, and all subsequent results are considered under-traced. Finally, record the number of participants involved in judgment and correction during the reconstruction process, and calculate the F1 score based on the final reconstruction results as the evaluation metric.

In conventional neuronal reconstruction simulation, the process differs from the precision approach. The conventional method employs a back-to-back approach, where three personnel independently reconstruct the entire neuron, with their reconstruction accuracy matched to that of the personnel in the precision simulation. For positions with consistent reconstruction results, the number of personnel involved is recorded; for positions with conflicts, the number of personnel working individually at different locations is recorded. For accuracy computation at conflict positions, the probability of each possible result being correct is calculated based on individual personnel results and accuracy, and a random value is used to select the final result. If the reconstruction result is correct, the reconstruction continues to extend; if the result is incorrect, a branch in the current data is randomly selected as the erroneous branch, and a random number is generated to determine its type (over-tracing or under-tracing). The handling method follows that of precise reconstruction. By repeating the above steps, simulated results of conventional neuronal reconstruction are obtained.

After obtaining the results from both reconstruction methods, their reconstruction efficiency and accuracy are compared to demonstrate the superiority of precision-assigned neuronal reconstruction over conventional methods.

## Results

### Results of precise data–algorithm matching reconstruction

This experiment includes datasets acquired from two imaging systems: HD-fMOST [34] and TDI-fMOST [35]. For each system, 4,000 fully reconstructed samples were selected. Each sample consists of a three-dimensional image dataset with variable size and the corresponding correctly reconstructed single-neuron skeleton. Among them, 2,000 samples were used to determine the optimal matching reconstruction methods, while the remaining 2,000 samples were used to validate the effectiveness of the proposed matching reconstruction algorithm.

**Fig. 2:**
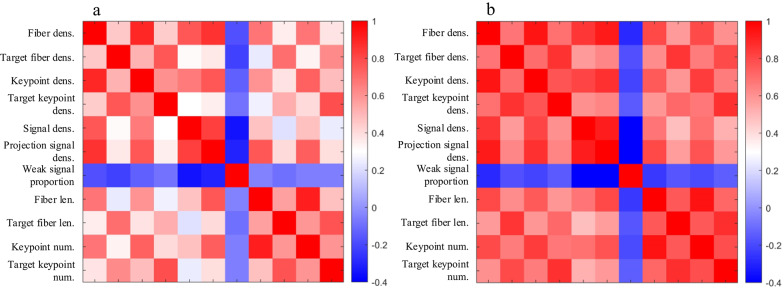
Correlation maps among different image difficulty indicators. (**a**) Correlation coefficient matrix of image features for data acquired by the HD-fMOST system. (**b**) Correlation coefficient matrix of image features for data acquired by the TDI-fMOST system

In the precise matching reconstruction process, different image metrics were grouped into categories according to the correlation coefficients among them. Figure 2(a) shows the correlation map of image metrics for data acquired by the HD-fMOST system. Metrics with correlation coefficients greater than or equal to 0.8 were considered to belong to the same category.

As shown in the figure, for data acquired by the HD-fMOST system, fiber density, key-point density, signal density, and projection signal ratio exhibit strong correlations with each other; therefore, these four image metrics were grouped into one category. In contrast, target fiber density, target key-point density, weak signal ratio, target fiber length, and the number of target key points were each treated as independent categories due to their weak correlations with other metrics. Finally, fiber length and the number of key points were grouped into one category.

Similarly, Fig. 2(b) illustrates the correlation matrix of image indicators for data acquired by the TDI-fMOST system. As shown in the figure, fiber density, keypoint density, fiber length, number of crossing points, signal density, and projection signal ratio demonstrate strong correlations and are therefore grouped into one category. Target fiber density, target keypoint density, target fiber length, and the number of target keypoints are also strongly correlated and are grouped into another category. The weak signal ratio, however, is treated as a separate category.

**Fig. 3:**
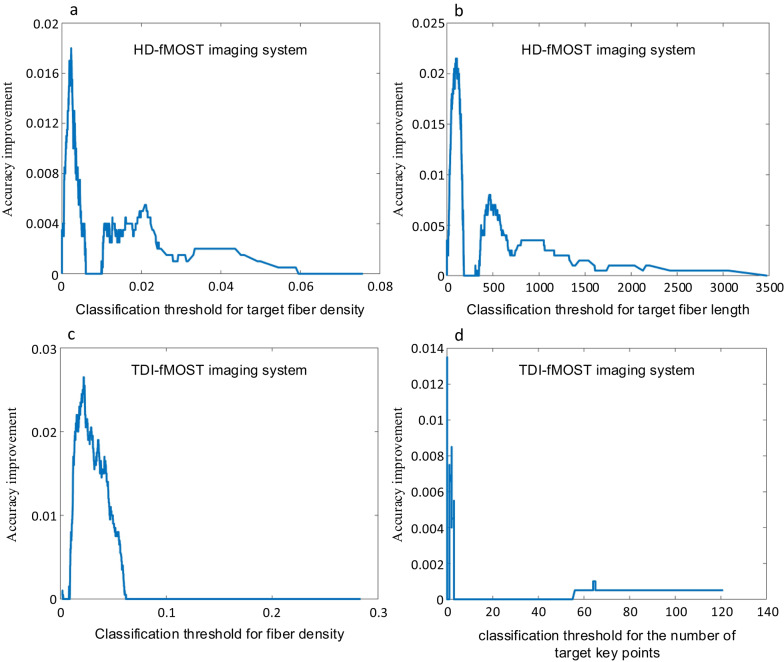
Accuracy improvement of neuronal reconstruction under different indicator threshold settings. (**a**) Effect of the classification threshold of target keypoint density on reconstruction accuracy. (**b**) Effect of the classification threshold of target fiber length on reconstruction accuracy. (**c**) Effect of the classification threshold of keypoint number on reconstruction accuracy. (**d**) Effect of the classification threshold of target keypoint number on reconstruction accuracy

After completing the indicator grouping, we further defined threshold values for each image indicator and gradually adjusted the threshold from the minimum to the maximum value of that indicator. For each threshold setting, the images were divided into two categories accordingly. Within each category, the automated reconstruction method with the highest accuracy was selected for reconstruction. The results were then compared with those obtained using a single optimal reconstruction algorithm, thereby quantifying the accuracy improvement brought by the classification-based matching strategy. Subsequently, indicators with marginal accuracy improvements were discarded, and only the two most effective indicators were retained. Figure 3(a-b) present the two key indicators finally selected for the HD-fMOST imaging system, namely target keypoint density and target fiber length. As shown in the figure, when target keypoint density was used as the classification criterion with a single threshold for image partitioning, and the optimal reconstruction algorithm was selected for each category, the overall reconstruction accuracy improved by 1.10%. When target fiber length was used as the classification indicator, the classification-based matching strategy led to an accuracy improvement of 2.20%.

Figure 3(c-d) present the two image classification indicators finally selected for the TDI-fMOST imaging system, namely keypoint number and target keypoint number. The results show that when keypoint number is used as the classification indicator with a single-threshold image partitioning, and the optimal reconstruction algorithm is applied to each category, the reconstruction accuracy improves by 4.10%. When target keypoint number is used as the classification indicator, the reconstruction accuracy increases by 3.15%.

**Fig. 4:**
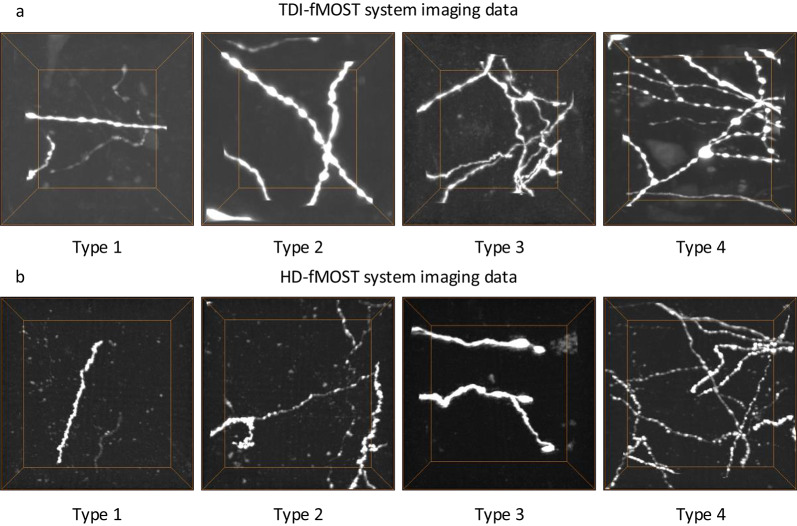
Neuronal data classification results

In summary, we selected the two indicators that have the greatest impact on reconstruction accuracy for image classification. For data acquired by the HD-fMOST system, target keypoint density and target fiber length were ultimately chosen as the classification indicators. For data acquired by the TDI-fMOST system, fiber density and target keypoint number were selected as the classification indicators. The image classification results are shown in Fig. 4, where Fig. 4(a) presents the classification results for HD-fMOST data, and Fig. 4(b) shows the classification results for TDI-fMOST data. Based on the classified images, a corresponding precision-matching reconstruction strategy was established to improve the overall accuracy of automated reconstruction.

Because the HD-fMOST and TDI-fMOST systems exhibit significant differences in imaging background characteristics, the selection of image classification indicators and the optimal method combinations must be analyzed and validated separately. For the HD-fMOST imaging data, we randomly selected 2,000 three-dimensional image blocks as a training set and used an exhaustive search approach to determine the optimal classification thresholds. The results show that the optimal classification threshold for target keypoint density is 0.0001, and the optimal threshold for target fiber length is 106.33. Based on these thresholds, HD-fMOST images are divided into four categories: the first category with target keypoint density < 0.0001 and target fiber length < 106.33; the second category with target keypoint density < 0.0001 and target fiber length $\ge$ 106.33; the third category with target keypoint density $\ge$ 0.0001 and target fiber length < 106.33; and the fourth category with target keypoint density $\ge$ 0.0001 and target fiber length $\ge$ 106.33.

**Table 1:** Reconstruction accuracy of different types of data in the HD-fMOST imaging system

Methods	Type 1 Images	Type 2 Images	Type 3 Images	Type 4 Images	Total
Single Method (Training Set)	0.319	0.200	0.666	0.177	0.532
Precision Matching (Training Set)	0.409	0.400	0.666	0.274	0.564
Single Method (Testing Set)	0.280	0.500	0.675	0.167	0.541
Precision Matching (Testing Set)	0.377	0.000	0.675	0.188	0.564
Improvement	0.094	0.000	0.000	0.061	0.028

**Fig. 5:**
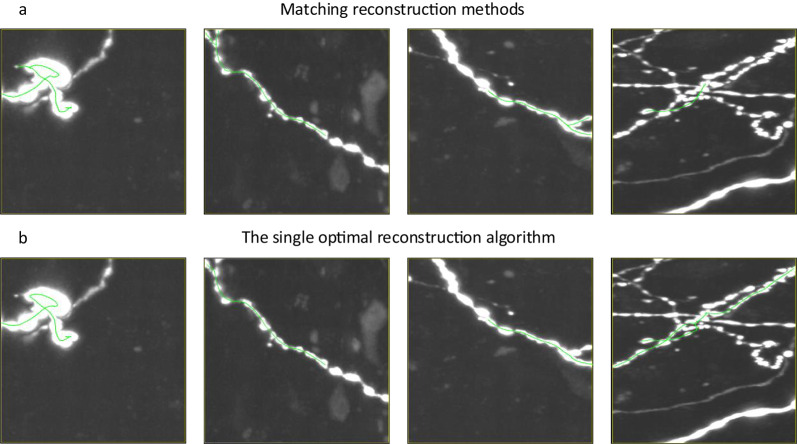
Comparison of precision matching reconstruction and conventional reconstruction results

**Fig. 6:**
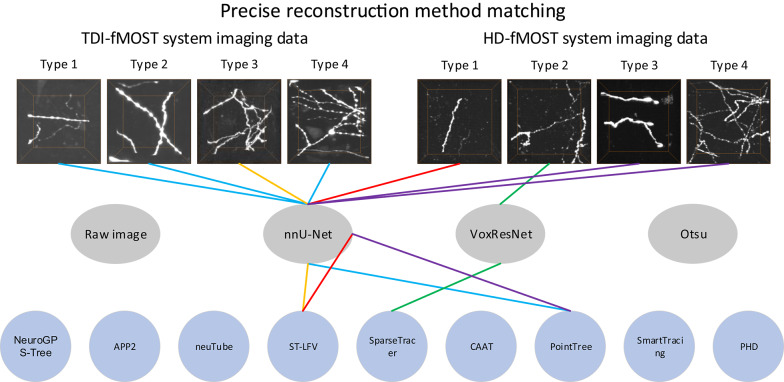
Matching results between different data types and reconstruction algorithms

Further analysis of the optimal method combinations for different categories revealed that for the first, second, and fourth categories, the optimal reconstruction method combination is nnU-Net for signal extraction and PointTree for reconstruction, whereas for the third category, the optimal combination is nnU-Net with ST-LFV, as shown in Fig. 6. Overall, the single optimal method combination is nnU-Net with ST-LFV. We first qualitatively analyzed the advantages and disadvantages of the precision-matching reconstruction method compared to the single optimal method. As shown in Fig. 5, the matching reconstruction algorithm achieves higher reconstruction accuracy than the single method in cases involving branching and complex structures. To further qualitatively validate the effectiveness of the precision-matching method, we randomly selected 2,000 three-dimensional HD-fMOST image blocks as a test set and compared the reconstruction performance of the precision-matching algorithm and the single optimal method on both the training and test sets. As shown in Table 1, the classification-based method significantly outperformed the single method across the two categories of images, with the largest improvement observed for the first category, achieving a 9.4% increase in automated reconstruction accuracy. The fourth category showed an accuracy improvement of 6.1%. Overall, the precision-matching reconstruction algorithm increased the overall accuracy by 2.8% compared to the single reconstruction algorithm. These results indicate that precision selection based on image classification can significantly enhance the automated reconstruction accuracy of HD-fMOST imaging data.

For the TDI-fMOST imaging data, the same approach was applied. A training set of 2,000 three-dimensional image blocks was randomly selected, and an exhaustive search was used to determine the optimal classification thresholds. The results showed that the optimal threshold for keypoint number is 3, and for target keypoint number it is 2. Based on these thresholds, TDI-fMOST images can also be divided into four categories: the first category with keypoint number < 3 and target keypoint number < 2; the second category with keypoint number < 3 and target keypoint number $\ge$ 2; the third category with keypoint number $\ge$ 3 and target keypoint number < 2; and the fourth category with keypoint number $\ge$ 3 and target keypoint number $\ge$ 2.

Further analysis revealed that the optimal reconstruction method combination for the first category of images is nnU-Net for signal extraction with ST-LFV for reconstruction, while the second category’s optimal combination is VoxResNet for signal extraction with SparseTrace for reconstruction. For the third and fourth categories, the optimal combination is nnU-Net with PointTree, as shown in Fig. 6. Overall, the single optimal method combination is nnU-Net with ST-LFV. To quantitatively assess the superiority of the precision-matching reconstruction approach, we randomly selected 2,000 three-dimensional TDI-fMOST image blocks as a test set and compared the reconstruction performance of the precision-matching algorithm with the single optimal method on both the training and test sets. As shown in Table 2, overall, the second to fourth categories of images achieved significant improvements compared with the single method, with the largest accuracy increase of 18.8% observed in the fourth category. The overall reconstruction accuracy increased by 4.6%. These results further demonstrate that for TDI-fMOST imaging data, the classification-based precision reconstruction approach can effectively enhance the accuracy of automated reconstruction.

**Table 2:** Reconstruction accuracy of different types of data in the TDI-fMOST imaging system

Methods	Type 1 Images	Type 2 Images	Type 3 Images	Type 4 Images	Total
Single Method (Training Set)	0.762	0.311	0.544	0.139	0.607
Precision Matching (Training Set)	0.762	0.356	0.619	0.314	0.650
Single Method (Testing Set)	0.792	0.364	0.510	0.127	0.630
Precision Matching (Testing Set)	0.792	0.386	0.595	0.330	0.674
Improvement	0.000	0.034	0.079	0.188	0.046

In summary, for both the HD-fMOST and TDI-fMOST systems, images are divided into four categories based on different difficulty indicators, providing a basis for matching each category with its optimal method combination. This strategy significantly improves overall reconstruction accuracy in both the training and test sets for the two systems, with particularly pronounced benefits for complex categories. These results indicate that the precision-matching algorithm not only enhances the accuracy of automated reconstruction but also substantially reduces subsequent manual correction and verification efforts, providing a feasible pathway for achieving high-quality, large-scale automated neuronal reconstruction.

### Precision-allocated neuronal reconstruction results

During the task allocation process for neuronal reconstruction, we conducted a systematic comparison to evaluate the performance of different optimization methods. By varying the total number of reconstruction participants, we analyzed the differences in objective values under different personnel scales and comprehensively compared multiple heuristic algorithms to validate the superior performance of the greedy algorithm in solving allocation problems.

**Fig. 7:**
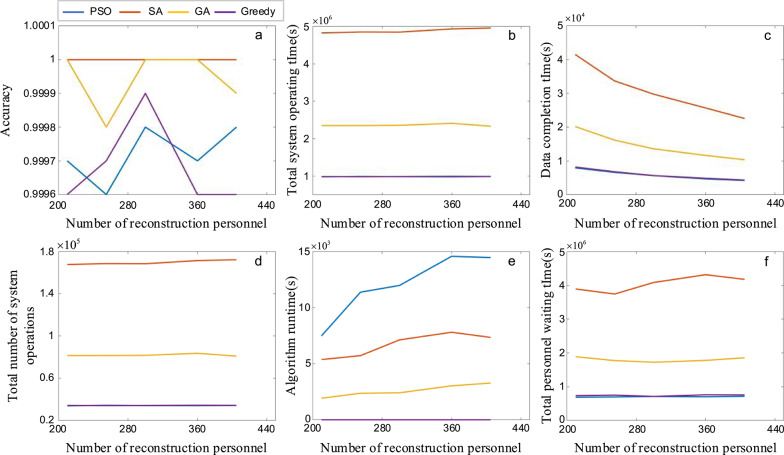
Performance comparison of different solution methods under varying reconstruction workforce sizes. (**a**) Accuracy comparison of different methods. (**b**) Comparison of total system completion time for different methods. (**c**) comparison of data completion time for different methods. (**d**) Comparison of total number of system operations for different methods. (**e**) Comparison of algorithm running time for different methods. (**f**) Comparison of total personnel waiting time for different methods

The results of data allocation and the performance of different solution methods are shown in Fig. 7(a-f). the results indicate that, as the number of workers increases, the overall reconstruction accuracy exhibits slight fluctuations, but all solution methods consistently meet the predefined accuracy requirement. Among all algorithms, the simulated annealing algorithm demonstrates the highest stability, achieving an accuracy of 1 under all tested conditions.

Regarding the total system operation time, variations in the number of workers have no significant impact. Particle swarm optimization and the greedy algorithm achieve the best performance in this metric, while the genetic algorithm and simulated annealing require comparatively longer computation time. For data completion time, the overall processing duration gradually decreases as the number of workers increases, with particle swarm optimization and the greedy algorithm again outperforming the others.

In terms of the total number of task executions within the system, the results are nearly unaffected by changes in personnel scale. Particle swarm optimization and the greedy algorithm show the best performance on this metric. Concerning computational efficiency, notable differences are observed among the algorithms: the greedy algorithm achieves the highest solving efficiency and remains largely unaffected by changes in personnel size, whereas particle swarm optimization demonstrates relatively lower solving efficiency. with respect to worker waiting time, particle swarm optimization and the greedy algorithm exhibit comparable performance, both outperforming the genetic algorithm and simulated annealing, and showing minimal sensitivity to variations in the number of workers.

Furthermore, to quantitatively evaluate the performance differences between the optimization methods, we conducted a t-test between the greedy algorithm and the particle swarm optimization (PSO) algorithm to determine whether there were significant differences across various performance metrics. The optimal objective values of the other two methods were significantly lower than those of the greedy and PSO algorithms, indicating their overall performance was comparatively weaker; therefore, no further quantitative comparisons were conducted for these methods. We calculated the t-test p-values for the greedy algorithm and PSO algorithm in terms of total system operation time, data completion time, and total number of task executions, which were 0.26, 0.88, and 0.29, respectively, all greater than 0.05, indicating no significant differences between the two methods for these metrics. However, in terms of computational efficiency, the greedy algorithm’s solving time was over 60,000 times faster than that of the PSO algorithm; the corresponding t-test p-value was 0.000015, far below 0.05, indicating a significant difference in computational efficiency. Therefore, the greedy algorithm can be considered to have a clear computational efficiency advantage in this task allocation optimization problem.

Overall, while the greedy algorithm and PSO show no significant differences in terms of objective function performance, they differ substantially in computational efficiency. Given its extremely low computational cost, the greedy algorithm is considered a more practical heuristic for neuronal reconstruction task allocation, offering a highly effective strategy to improve task allocation efficiency while ensuring reconstruction accuracy.

### Performance comparison of precision-assigned reconstruction

To compare the performance of the precise allocation reconstruction method with that of the conventional reconstruction method, we selected 10 reconstructed mouse brain datasets for simulation, thereby qualitatively analyzing the differences between the proposed precise allocation reconstruction strategy and the conventional approach. Each dataset included one data block, the reconstruction results of each processing step, the corresponding difficulty level of the data, and the correctly reconstructed single-neuron skeleton structure.

**Fig. 8:**
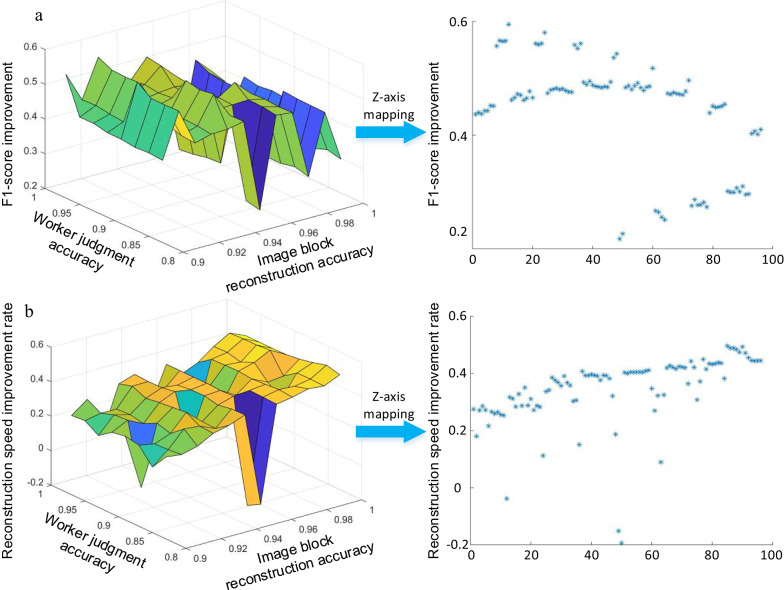
Performance comparison between precision-assigned reconstruction and conventional reconstruction. (**a**) Improvement in F1-score of precision-assigned reconstruction relative to conventional reconstruction. (**b**) Speed-up ratio of precision-assigned reconstruction relative to conventional reconstruction

During the analysis, reconstruction accuracy and efficiency were calculated under different evaluator judgment accuracy and data block reconstruction accuracy settings to validate the effectiveness of the precision allocation reconstruction method under varying conditions. Specifically, evaluator judgment accuracy was varied from 0.8 to 1.0, data block reconstruction accuracy was varied from 0.9 to 1.0, correction accuracy was fixed at 0.85, and automatic reconstruction accuracy was determined from actual measured results.

Under these settings, the reconstruction process of real neuronal data was simulated to compare the reconstruction accuracy of the conventional method with that of the precision allocation reconstruction method, as shown in Fig. 8(a). In the left picture, the Z-axis represents the accuracy improvement of the proposed method relative to the conventional method. The results show that, across different evaluator judgment accuracy and data block reconstruction accuracy settings, the precision allocation reconstruction strategy consistently outperformed the conventional method. Specifically, the conventional method achieved an average F1-score of 47.8%, whereas the precision allocation method achieved an average F1-score of 92.0%, corresponding to an average improvement of 44.3%. To intuitively observe the accuracy improvement of each data point, we projected the three-dimensional data points onto the z-axis for analysis. The results show that the F1-score improvement of all data points exceeds 0.2, indicating that the precision-matching reconstruction method significantly outperforms the conventional reconstruction method in terms of reconstruction accuracy.

Furthermore, to determine whether the difference in reconstruction accuracy between the two methods was statistically significant, an independent-samples t-test was performed on the F1-scores obtained by the two approaches. The results yielded a p-value of $5 \times {10^{ - 106}}$, which is far below the significance threshold of 0.05, indicating a statistically significant difference between the two methods. Therefore, the proposed precise allocation reconstruction method can be considered significantly superior to the conventional method in terms of reconstruction accuracy, further validating its effectiveness and superiority from a statistical perspective.

We also compared the reconstruction efficiency of the two methods under different conditions. The efficiency evaluation was calculated using the following formula:

9 $$\begin{aligned} {R_t} = \frac{{{T_n} - {T_u}}}{{{T_n}}} \end{aligned}$$

Here, ${T_n}$ represents the time required by the conventional reconstruction method, while ${T_u}$ denotes the time required by the accuracy-model-based reconstruction method. The results indicate that the precision allocation reconstruction method based on the accuracy model significantly outperforms the conventional method in terms of reconstruction efficiency. A positive evaluation value indicates an acceleration advantage.

Figure 8(b) presents the overall efficiency improvement achieved by the precision allocation reconstruction method relative to the conventional reconstruction method. The results show that the precision allocation strategy is more efficient in most scenarios, yielding an average speed improvement of 34.6%. To visually assess the improvement in reconstruction speed for each data point, we projected the three-dimensional data points onto the z-axis for analysis. The results show that only three points experienced a slight decrease in speed, while the reconstruction efficiency of all other points improved, indicating that the precision-matching reconstruction method significantly outperforms the conventional method in terms of reconstruction efficiency.

In addition, an independent-samples t-test was conducted on the reconstruction times obtained by the two methods to determine whether the observed difference in efficiency was statistically significant. The calculated p-value was $5 \times {10^{ - 70}}$, which is far below the significance threshold of 0.05, indicating a statistically significant difference in reconstruction efficiency between the two methods. These results demonstrate that the proposed precision allocation method can significantly improve reconstruction efficiency.

In summary, the precision allocation neuronal reconstruction method outperforms the conventional approach in both reconstruction accuracy and efficiency. The proposed method not only ensures high reconstruction quality but also significantly accelerates the reconstruction process, providing a reliable and efficient solution for large-scale neuronal reconstruction.

## Discussion

This study proposes a quantitative precision reconstruction strategy based on neuronal data grading. By systematically analyzing the core challenges in neuronal reconstruction, we established models for reconstruction efficiency and accuracy, and quantitatively evaluated the influence of various factors on reconstruction quality and efficiency. The results indicate that the judgment accuracy of human operators has the greatest impact on reconstruction quality, whereas the accuracy of automated reconstruction plays a key role in improving reconstruction efficiency. These findings provide a theoretical basis for subsequent precision neuronal reconstruction.

To address the challenges posed by the complex structure of neurons and the diversity of signals, where a single method cannot handle all cases effectively, we proposed a precision matching strategy of “data–signal extraction–automated reconstruction method.” For different categories of data, the optimal combination of signal extraction methods and automated reconstruction algorithms was selected, ensuring that the automated reconstruction accuracy of each data category reached its optimum. This approach significantly improved reconstruction accuracy across multiple data types, with a maximum improvement of 18.8%, thereby laying a foundation for large-scale neuronal reconstruction.

To address the diversity of data and variations in annotator expertise, we further developed a precision-assigned optimization model for neuronal reconstruction. This model enables accurate matching between data of varying difficulty and the capabilities of annotators. By ensuring high reconstruction quality, the model allows neuronal reconstruction to be completed with minimal time and cost, while simultaneously balancing reconstruction quality and efficiency, providing technical support for large-scale neuronal reconstruction.

To validate the effectiveness of this method, we conducted simulation experiments based on real reconstruction data, comparing the precision-assigned reconstruction process with conventional reconstruction. Reconstruction time and accuracy were recorded. The results show that precision-assigned reconstruction significantly outperforms conventional methods in accuracy, while both judgment and correction times are substantially lower, resulting in a marked improvement in overall efficiency. Compared with conventional methods, the precision-assigned strategy increased reconstruction accuracy by 44.3% and overall efficiency by 34.6%. This further demonstrates that the proposed method can effectively support large-scale, high-quality neuronal reconstruction.

Despite these positive results, certain limitations remain. First, the data grading method employed is not fully automated and still requires some manual intervention. Second, although the precision matching strategy improves automated reconstruction accuracy, it still falls short of achieving fully automated reconstruction. Finally, while this study conducted a preliminary analysis of factors affecting reconstruction efficiency and accuracy, the optimization strategies are not yet comprehensive. In order to further improve the quality and efficiency of reconstruction, deep learning methods can be used to finely allocate data and construct an optimal reconstruction method for different types.

## Conclusion

In summary, this study proposes a quantitative and precision-oriented neuronal reconstruction method, enabling precise control over the reconstruction process and ensuring high-quality outcomes. By implementing precise matching between data and algorithms and integrating the performance advantages of multiple reconstruction methods, the approach achieves accurate alignment between different types of data and their corresponding reconstruction methods, effectively improving the accuracy of automated reconstruction and overall reconstruction efficiency. Furthermore, a precision-assigned optimization model based on data grading allows reconstruction tasks to be completed with minimal time and cost while maintaining high reconstruction quality. Comparative experiments further validate the effectiveness and superiority of the precision-assigned strategy. Overall, this study provides reliable technical support for large-scale, high-quality neuronal reconstruction and establishes a solid data foundation for subsequent neuroscience research.

## Data Availability

The code for neuron precision-matching reconstruction and neuron precision-allocation reconstruction is available at the following link: https://github.com/Brainsmatics/pMatch_code.
